# Antarctic Climate Change: Extreme Events Disrupt Plastic Phenotypic Response in Adélie Penguins

**DOI:** 10.1371/journal.pone.0085291

**Published:** 2014-01-29

**Authors:** Amélie Lescroël, Grant Ballard, David Grémillet, Matthieu Authier, David G. Ainley

**Affiliations:** 1 Biodiversité et gestion des territoires, URU 420, Université de Rennes 1, Rennes, France; 2 Centre d'Ecologie Fonctionnelle et Evolutive, CNRS, UMR 5175, Montpellier, France; 3 Point Blue Conservation Science, Petaluma, California, United States of America; 4 Percy FitzPatrick Institute and DST/NRF Excellence Centre, University of Cape Town, Rondebosch, South Africa; 5 H.T. Harvey & Associates, Los Gatos, California, United States of America; Norwegian Polar Institute, Norway

## Abstract

In the context of predicted alteration of sea ice cover and increased frequency of extreme events, it is especially timely to investigate plasticity within Antarctic species responding to a key environmental aspect of their ecology: sea ice variability. Using 13 years of longitudinal data, we investigated the effect of sea ice concentration (SIC) on the foraging efficiency of Adélie penguins (*Pygoscelis adeliae*) breeding in the Ross Sea. A ‘natural experiment’ brought by the exceptional presence of giant icebergs during 5 consecutive years provided unprecedented habitat variation for testing the effects of extreme events on the relationship between SIC and foraging efficiency in this sea-ice dependent species. Significant levels of phenotypic plasticity were evident in response to changes in SIC in normal environmental conditions. Maximum foraging efficiency occurred at relatively low SIC, peaking at 6.1% and decreasing with higher SIC. The ‘natural experiment’ uncoupled efficiency levels from SIC variations. Our study suggests that lower summer SIC than currently observed would benefit the foraging performance of Adélie penguins in their southernmost breeding area. Importantly, it also provides evidence that extreme climatic events can disrupt response plasticity in a wild seabird population. This questions the predictive power of relationships built on past observations, when not only the average climatic conditions are changing but the frequency of extreme climatic anomalies is also on the rise.

## Introduction

Coincident with rapid global climate change, the frequency of extreme events (e.g. heat waves, precipitation, storm surges) is expected to increase in many regions [1], [2], [3]. In support, a recent study showed that the frequency of hot temperature anomalies has been increasing worldwide in the past 30 years [4]. Extreme climatic events can trigger dramatic biotic responses at different levels of ecological organisation, from the individual (e.g. physiological stress) [5] to the ecosystem (shifts between states) [6], [7]. It is therefore increasingly acknowledged that extreme climatic events could play a more important role in shaping population and community dynamics as well as species distribution than changes in average climatic conditions [8], [9]. Faced with extreme climatic events, animals may die [10], or move [11], sometimes leading to local extinctions and range contractions [12]. However, extreme climatic events do not necessarily translate into extreme ecological responses as some populations may be able to adapt and avoid survival costs [13], [14].

In polar regions, where global circulation models predict more pronounced warming than in temperate and tropical regions [15], increasingly warm subsurface ocean temperatures [16] [17] are already triggering intensified ice-sheet loss through basal melting of ice shelves [18], [19]. For benthic invertebrate communities [20], as well as for vertebrate mesopredators [11], [21], the calving and subsequent grounding of large icebergs from ice shelves certainly constitute extreme events in terms of reduction of vital rates, direct habitat destruction and indirect alteration of sea ice and primary production dynamics [22], [23], although in some species, the short-term impacts can be modest [14].

In Antarctica, while net sea ice cover (i.e., the area of ocean covered by ice) has increased over the past few decades owing to wind changes brought largely by mid-latitude warming and the Antarctic Ozone Hole [24], [25], modelled predictions point to a decrease by 5–15%, depending on sector, by 2025–2052 [26]. Concomitantly, increased ice shelf instability [19], [27] will lead to more frequent iceberg calving [20], including very large (hundreds of km^2^) icebergs [28]. Both changes in average sea ice conditions and frequency of extreme events are likely to affect pagophilic species.

To date, studies that have addressed the impact of climate change on Antarctic seabird populations have mainly focused on linking climate variability and demographic parameters of populations [29], [30] and references therein, [31]. However, mechanisms underlying such patterns occur at the individual level, as it is the individual who must deal with the changing environment. In long-lived species especially, phenotypic plasticity, i.e. the ability of an organism to express different phenotypes depending on the environment [32], is often regarded as central in the ability of organisms to respond to rapidly changing conditions and increased uncertainty, e.g. [33].

The Adélie penguin (*Pygoscelis adeliae*) depends in a complex way on sea ice for foraging, resting, moulting and migrating [34], [35]. Indeed, Adélie penguins feed on epontic species, i.e. species that live on the underside of sea ice (e.g. Antarctic krill *Euphausia superba* and crystal krill *Euphausia crystallorophias*), as well as on predators of these species (e.g. Antarctic silverfish *Pleuragramma antarcticum*). Adélie penguins can also use sea ice as a physical barrier to trap their prey [36]. They are therefore dependent year-round on sea ice as a foraging habitat. Ice floes are also used by Adélie penguins to rest (all year-round) and moult (in February, immediately after the breeding season) out of reach of their predators, i.e. leopard seals *Hydruga leptonyx* [34], [37] and possibly killer whales *Orcinus orca* in limited areas [38], R. Pitman, pers. comm., but also to ride the oceanic currents at minimal energetic costs during the autumn migration (March to June), from their coastal breeding grounds to the northern ice edge [39], [35], [40]. However, while sea ice is the foraging and resting habitat of Adélie penguins, too extensive and concentrated sea ice nearshore (tens of kilometres from the coast) during chick provisioning (December–January, when sea ice cover is minimal) will result in longer, more energetically costly foraging trips, lower chick feeding frequencies and ultimately lower breeding success as breeding adults have to walk, rather than swim, on large stretches of consolidated ice [34], [41], [42]. On a geologic scale, periods of extensive fast ice can lead to colony extinction [43]. In a related way, the existence of polynyas (i.e. areas within the region of ice cover that are ice-free or persistently have significantly lower ice concentration than the surrounding pack) in the vicinity of the breeding colonies is critical, as they reduce the commuting time and energy expenditure between colony and food supply [34], [26]. At a larger spatial scale (hundreds of kilometres), too extensive offshore sea ice will also impede the spring migration (August to October) from the northern wintering grounds to the southern coastal breeding grounds and results in poor body condition and delayed breeding [34], [42], [44]. In summary, Adélie penguins need pack ice all year round at the large scale (hundreds of kilometres) and open water near their breeding colonies during summer at the mesoscale (tens of kilometres), but they are negatively affected by extensive, consolidated ice at any spatial scale.

If summer pack ice disappears in the northernmost Antarctic regions, Adélie penguins will be forced to reduce their geographic range southwards, as is occurring in the rapidly warming western Antarctic Peninsula region [45]. The Ross Sea, which features the southernmost marine habitat on Earth and already harbours 38% of the world Adélie penguin populations [46], might then become the last refuge for Antarctic penguins [26]. It is therefore of prime importance to determine the capacity of individual penguins to respond to Antarctic sea ice cover variability, so as to evaluate how they will cope with forecasted changes in their habitat.

In January 2001, the grounding of two giant icebergs, B15-A (ca. 140×30 km) and C16 (ca. 50×25 km), against Ross Island in the south-western Ross Sea significantly affected the sea ice dynamics, primary productivity and demographic parameters of several mesopredator species. These icebergs restricted the normal drift of pack ice in the western Ross Sea, resulting in higher spring/summer ice cover (ice per unit area) and a drastic reduction in regional primary productivity [22], [47], though the effect on primary productivity lasted only during the initial year (C. Smith, unpubl. data). These icebergs remained until July 2005, and had variable effects on upper-trophic level species. For Emperor penguins *Aptenodytes forsteri*, the collision of B15-A with the Ross Ice Shelf caused the death of numerous incubating adults and the induced changes in nesting habitat reduced chick production (0–40% of the chick production in pre-iceberg years) over the long-term [48]. For Weddell seals *Leptonychotes weddellii*, the icebergs prevented the breakout of what is normally annual fast ice for five years in McMurdo Sound, thus thickening the ice and reducing the availability of ice cracks needed for ocean access; in turn, a large portion of the population temporarily vacated the area and those that remained demonstrated decreased pupping/recruitment success but not decreased survival rates [49], [14]. For Adélie penguins, the adult survival rates were similarly unaffected [50], [11] but iceberg presence in the vicinity of their breeding colonies resulted in increased dispersal rates [51], [11], longer foraging trips, less food delivered to chicks [44], lower chick feeding frequency [21] and lower chick production (<0.20 compared to 0.80–1.36 chicks per pair during pre-iceberg years 1997 to 2000; [52], D.G. Ainley, G. Ballard & K. Dugger, unpublished). Such changes brought by the iceberg natural experiment help to reveal the mechanisms by which Adélie penguins cope with altered sea ice cover over the immediate and geologic time scales [43].

While Ballard et al. [44] showed that, in the Southern Ross Sea, (1) up to 12% sea ice concentration (SIC, % ocean area covered by ice) at the mesoscale coincided with shorter foraging trips and more food delivered to Adélie penguin chicks, (2) and that the increased SIC resulted in longer trips and less food delivered to the chicks, they did not assess the effect of the presence of giant icebergs on the specific shape of the reaction norm [53] for foraging efficiency along a gradient of SIC. Yet it has been suggested that, rather than only modulating the average level of traits, extreme events could disrupt the plastic response [54], [55], [56]. In the context of predicted alteration of sea ice cover and increased frequency of extreme events, as well as understanding penguin response to changes in SIC in the past [43], it is therefore especially important to understand how extreme events can modify the relationship between foraging efficiency and SIC. Mega-icebergs are thought to have occurred in the Ross Sea about 20 times per millennium during the late Holocene [57], [58] when they would have blocked access to much of the usual penguin foraging area for a few years, changed local SIC and penguins' ocean accessibility [21], and thus are considered an extreme climate event in the current era [1].

Here, using longitudinal data collected over 13 years at Cape Crozier on Ross Island, we investigated the within- and between-individual variation in foraging behaviour of Adélie penguins in response to varying SIC in their foraging area. We tested the hypothesis that significant levels of behavioural phenotypic plasticity allow Adélie penguins to forage efficiently under a wide range of SIC. Additionally, the iceberg natural experiment during 5 consecutive years [51] allowed us to test the effects of extreme events on the relationship between foraging efficiency and SIC.

## Materials and Methods

### Ethics statement

All penguin survey, capture and handling methods performed during data collection for this study were approved under Antarctic Conservation Act permits (# 1997-010, 2000-007, 2003-002, 2006-010), issued by the US National Science Foundation and the U.S. Antarctic Program; and Institutional Animal Care and Use Committee permits issued by Oregon State University's (ACUP # 3049, 3672, 4130). Studies did not involve endangered species.

### Data set

**Figure 1:**
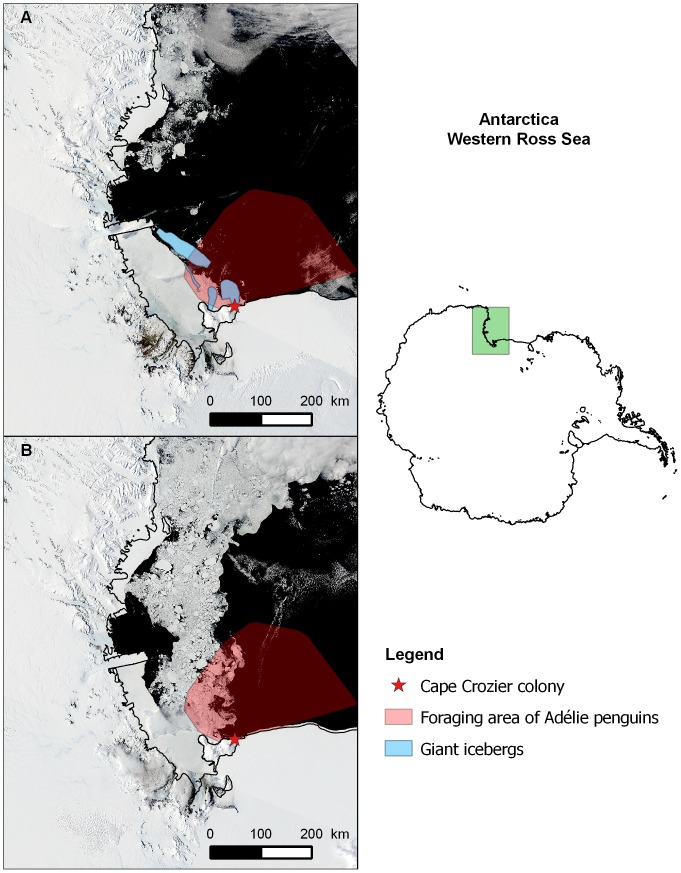
Map of the study area indicating the colony location (star), the foraging area of Adélie penguins (in red) and the location of giant icebergs. The satellite images are from http://lance-modis.eosdis.nasa.gov and illustrate A: a typical iceberg year (Dec. 21, 2004), B: a typical non-iceberg year (Dec. 21, 2008). The foraging area was determined as the polygon that contained 95% of at-sea positions of provisioning parents as determined by radio and satellite telemetry from 1997/1998 to 2008/2009.

**Figure 2:**
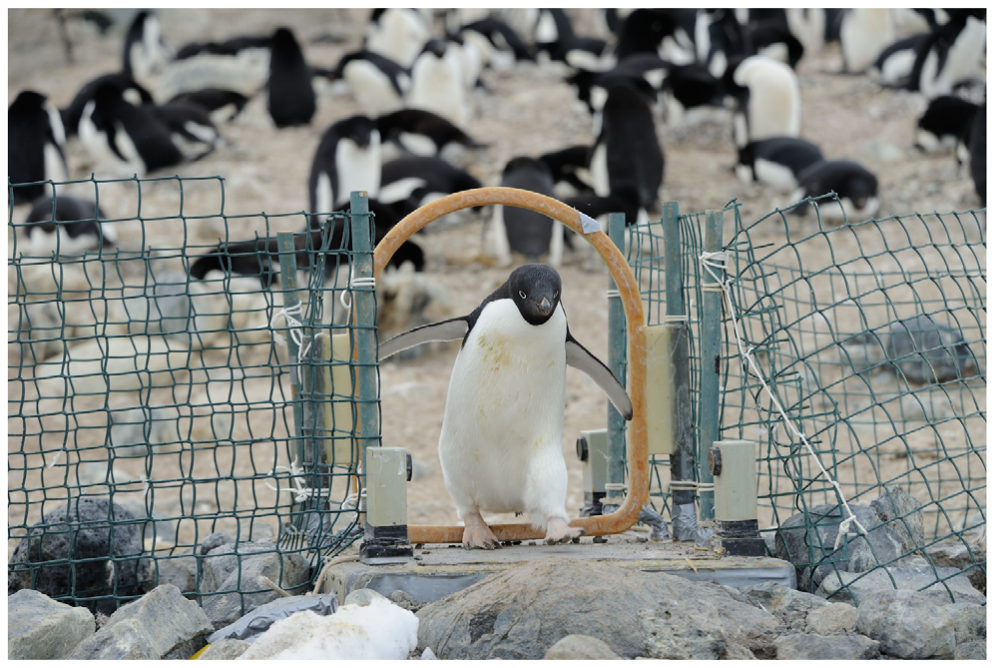
Adélie penguins are identified and weighed each time they cross the automated weighbridge on their way to/from the sea. Picture from David Grémillet.

Data on the foraging behaviour of chick-rearing Adélie penguins were collected from Dec 21 to Jan 7 each year from 1997/1998 to 2009/2010 at Cape Crozier (77°27′S, 169°12′E; ∼164 000 pairs; Fig. 1) using an automated weighbridge [59] (Fig. 2). This system recorded the date, time, direction, body mass and identification for each crossing of 22 to 74 breeding penguins per year, implanted with a radio frequency identification (RFID) tag. Some were also banded on the left flipper [60], and the potential effect of banding on foraging efficiency was statistically taken into account by including the type of marking as a fixed effect in the full starting model. Over the 13 years of study, we collected data from a total of 229 individuals (22 to 74 each year), who performed 1,759 provisioning trips. Each bird was followed within the breeding season, and over multiple breeding seasons. On average, there was a total of 7.7 foraging trips per individual, ranging from 1 to 42 trips (median: 6).

Individual catch per unit effort (CPUE), an index of foraging efficiency [21], was calculated by dividing grams of food returned by the time required to collect it (minutes away). The quantity of food returned was calculated from weighbridge data as the mass that an individual parent weighed when entering the nesting area after a trip at sea minus its mass when it left the nesting area for a new foraging trip. This is a measure of how much food a parent brought back to the colony and includes the food both delivered to chicks and that digested by the parent while attending the nest. Birds were primarily sexed by copulatory position, but we also employed a combination of size (especially head and bill) and behavior cues (e.g. timing of arrival to the colony) to discriminate sexes [34]. As we were interested in the direct effect of SIC on foraging behaviour, daily SIC data derived from passive microwave imagery [61] were extracted for the penguin foraging area determined by telemetry [44]between 1997/1998 and 2008/2009 (Fig. 1). No telemetry data were collected in 2009/2010.

Therefore, each bird was associated with a sex, a type of marking (RFID tag only or RFID tag and band) and a number of years since marking. Each trip of a given bird was associated with a year, 1997–2010, the presence or absence of giant icebergs (present between 2001/2002 and 2005/2006 austral summers), a day within the year, a CPUE and a measure of the mean SIC within the foraging area during the trip.

The dataset used for this paper is deposited at the California Avian Data Center, available at http://data.prbo.org/apps/penguinscience/alldata/PhenotypicPlasticity2012/ (requires user registration and compliance with Point Blue Conservation Science data sharing policy).

### Behavioural reaction norms

Within- and between-individual variation in CPUE in response to varying SIC was described using behavioural reaction norms [62]. The simplest type of behavioural reaction norm describes a linear relationship between the behavior of an individual and a mean-centered environmental covariate. If a regression line is fitted to this relationship, the elevation will represent the response value exhibited in the average environment and the slope will be a measure of the behavioural plasticity of the individual. With multiple individuals, variation in individual elevation and/or slopes can be specified by fitting a random intercept and/or random slope, respectively, in a linear mixed model. Although very few studies have attempted to describe and quantify non-linear reaction norms in the wild (but see [63] for seabirds), such relationships can be estimated by including extra parameters in the regression equation, thereby partitioning linear and non-linear components of the reaction norm slope [64].

Under the hypothesis that different individual Adélie penguins might prefer different SIC in their foraging areas, we fitted behavioural reactions norms of the following form:
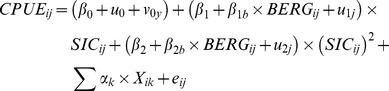
where CPUE_ij_ is the value of CPUE of measurement i for individual j, β_0_ is the average elevation, u_0j_ is the random intercept allowing variation in individual elevation, v_0y_ is the random intercept allowing variation in elevation depending on year (included to account for any sources of environmental covariance above that explained by SIC), β_1_ is the average slope and β_2_ is the average quadratic term for the dependence of CPUE_ij_ on SIC_ij_, β_1b_ and β_2b_ are additional average slopes depending on the presence/absence of icebergs (BERG). Knowing that extensive, consolidated ice is detrimental to these penguins, we included a quadratic term to model the dependence of CPUE on SIC. The coefficients u_1j_ and u_2j_ represent the phenotypic deviation of individual j from the population mean in each environment. ∑ α_k_ * X_ik_ are additional fixed effects and e_ij_ is the residual error term. CPUE was log-transformed so as to constrain the model predictions to positive values. SIC and other continuous fixed effects were standardized to improve model convergence. The models were fitted in R 2.14.2 [65] using packages {nlme} [66] for comparing models with and without random effects and {lme4} [67] for fitting models with crossed random effects.

### Model selection

Starting from a model with a full fixed effect structure including the additive effects of SIC, sex, type of marking (Mark), number of years since marking (TSM), BERG and study day (Day), as well as a quadratic effect of SIC (SIC^2^) and two interactions between SIC and BERG and between SIC^2^ and BERG, we tested the statistical significance of year and bird identity (ID) as random effects by comparing models with different random effect structures, using restricted maximum likelihood (REML) estimation [68]. Models were compared using log likelihood ratio tests following a forward procedure. As log likelihood ratio tests applied to mixed effects models are notoriously too conservative (i.e. they give p-values about twice as large as they should be [69]), we used a mixture of chi-square statistics (with equal weights of 0.5) as the limiting distribution of the log likelihood ratio test under the null hypothesis for estimating the associated p-values [70]. This method gave similar results as parametric bootstrapping (n = 1000 iterations) and greatly reduced computation time [71]. Model selection for random effects was also checked with Stochastic Search Variable Selection [72] which yielded the same results as the forward procedure.

Once the best random effect structure was selected, we chose the best fixed effect structure by iteratively comparing the full model with a nested model where the term exhibiting the lowest t-value was dropped, using maximum likelihood (ML) estimation. Then, the best model was fitted using REML estimation [68]. For assessing the quality of the model fit, we calculated r_c_, a goodness-of-fit statistic for non-linear mixed effects models, directly interpretable as a concordance correlation coefficient between observed and predicted values [73]. Model selection for fixed effects was checked with the LASSO [74] which yielded similar results.

Means are indicated ± s.e. unless noted.

## Results

Accounting for all fixed effects, including ID as a random effect, on the intercept significantly improved the model (LR = 106.3, p<0.001; Table 1), implying that individuals differed in their average level of efficiency (between-individual variation: 0.19 to 0.65 g min^−1^or 276 to 935 g day^−1^, depending on individuals). Including year as a random effect on the intercept also significantly improved the model (LR = 165.0, p<0.001), 2000 being the best year in terms of CPUE (+0.24 g.min^−1^) and 2007 the worst year (−0.14 g.min^−1^). However, models including a random slope per individual were not supported (LR = 4.06, p = 0.060) although we corrected for overly conservative p-values and double-checked our results using a Bayesian method: Stochastic Search Variable Selection (see Methods). Hence, there was no statistical evidence that individuals differed in how they responded to SIC (within-individual variation). Nonetheless, given the relatively low p-value that we obtained, these results will need to be confirmed by further studies.

**Table 1:** Forward model selection procedure for the random effect structure.

Model	Random intercept	Random slope	Log lik.	d.f.	Models compared	LR	Estimatedp-value
1	ID	-	590.25	12			
2	year	-	619.60	12	1 vs. 2	47.86	**<0.001**
**3**	ID+year	-	669.60	13	2 vs. 3	97.99	**<0.001**
4	ID+year	SIC	671.72	15	3 vs. 4	4.06	0.060
5	ID+year	SIC+SIC^2^	673.29	18	3 vs. 5	6.83	0.097

All models had the same fixed effects structure and were fitted with REML estimation. The fit of each successively more complex model was assessed using likelihood ratio tests. In models 4 and 5, random slopes per individual were modeled in addition to the random intercepts. LR refers to log-likelihood ratio test statistics.

**Figure 3:**
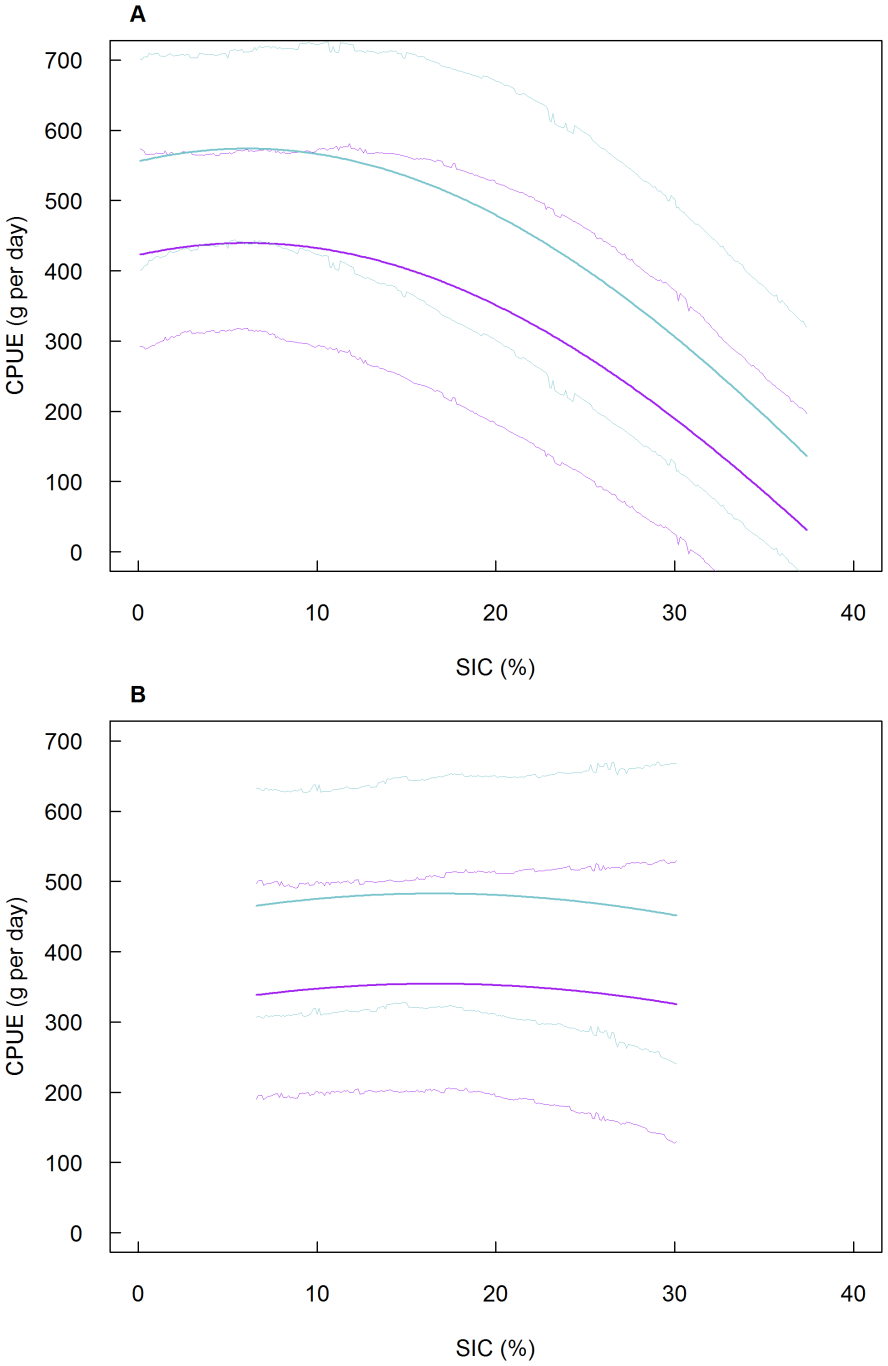
Predicted foraging efficiency (CPUE) of chick-rearing Adélie penguins depending on sea ice concentration. (a) Under “normal” environmental conditions, (b) under extreme (presence of giant icebergs) environmental conditions. Purple and blue lines represent values for females and males, respectively. Thick lines represent the average CPUE for each sex. Thin lines represent 95% Highest Posterior Density intervals computed from the posterior distribution of parameter estimates. Predictions were calculated from the following model: log (CPUE+1) = 0.267+0.069×sex (male)−0.051×Iceberg (yes)−0.010×SIC+(−0.074+0.075×Iceberg (yes))×SIC^2^.

The best fixed effect structure included the effects of sex, SIC, SIC^2^, BERG and an interaction between BERG and SIC^2^ (Table 2, r_c_ = 0.50). While the effect of SIC in itself was not significant (unlike SIC^2^), we retained it in our final model so as not to constrain the optimal SIC value to 0. For all individuals, SIC had a quadratic effect on foraging efficiency but the shape of this response differed in years of extreme conditions (i.e. presence of icebergs) compared to normal years (LR = 5.17, p = 0.023). Males were more efficient foragers (+0.07 g min^−1^, Table 3) than females (LR = 33.02, p<0.001). In normal years, Adélie penguins exhibited significant levels of plasticity in foraging efficiency depending on SIC variations. Maximum CPUE occurred for relatively low SIC, peaking at 6.1%, but was essentially the same from 0 to 12% and decreased with higher SIC (Fig. 3a). In years of extreme conditions, foraging efficiency did not vary much over the range of experienced SIC (6.6 to 30.1%). Maximum foraging efficiency peaked at 16.5% SIC, and was constrained to lower levels than under “normal” conditions (from 438.5±64.1 g day^−1^ on average for females under “normal” conditions to 353.7±78.2 g day^−1^ and from 573.1±68.8 g day^−1^ on average for males under “normal” conditions to 482.1±83.4 g day^−1^ under extreme conditions; Fig. 3b).

**Table 2:** Backward model selection procedure for the fixed effect structure.

Model	Dropped term	Log lik.	d.f.	Models compared	LR	p-value
3		718.52	13			
6	BERG : SIC	718.02	12	3 vs. 6	1.01	0.316
7	TSM	717.44	11	6 vs. 7	1.16	0.281
8	Mark	716.34	10	7 vs. 8	2.19	0.139
**9**	Day	714.77	9	8 vs. 9	3.15	0.076
10	BERG : SIC^2^	712.18	8	9 vs. 10	5.17	**0.023**
11	sex	698.26	8	9 vs. 11	33.02	**<0.001**

All models had the same random effects structure (ID and year as random intercepts) and were fitted with ML estimation. The full model (Model 3) included the following variables as fixed effects: SIC, SIC^2^, sex, Mark, TSM, BERG, Day, BERG : SIC, BERG : SIC^2^. The fit of each successively less complex model was assessed using likelihood ratio tests. LR refers to log-likelihood ratio test statistics.

**Table 3:** Output of the best model fitted using REML estimation and standardized variables.

Best model: 9			
	Intercept	Variance	SD
Random effects:	ID	0.004	0.060
	year	0.008	0.091
	Residual	0.023	0.152

		Estimate	SE	t-value
Fixed effects:	Intercept	0.267	0.036	7.507
	SIC	−0.010	0.046	−0.221
	SIC^2^	−0.074	0.025	−2.963
	sex	0.069	0.012	5.950
	BERG	−0.051	0.055	−0.919
	BERG : SIC^2^	0.075	0.029	2.572

In the final model, consistent differences between individuals accounted for 11% of the total variation in CPUE, while between-years differences accounted for 23% (Table 3). Residual variation remained the largest component, accounting for 66% of total variance.

## Discussion

Behavioural and physiological plasticity is recurrently seen as a major component of immediate strategies for coping with climate change [75], whose pace often exceeds the potential evolutionary response of species to selection [76]. Here, we show that ice-dependent Adélie penguins exhibit significant levels of behavioural plasticity in response to SIC variability, which in theory should allow them to cope with lower summer SIC in their southernmost habitat. In addition, our results provide field-based evidence that this plastic response can be disrupted by extreme events, questioning both (1) the accuracy of functional relationships and climate envelop models extrapolating from historical observations [77], as well as (2) the role of phenotypic plasticity in the adaptation of species to climate change [55].

### Plasticity of foraging efficiency

The plasticity of a complex phenotype such as parental provisioning behaviour has rarely been described (but see [78]). Here, we show that under normal environmental conditions (i.e., in absence of relatively infrequent calving of giant icebergs from the Ross Ice Shelf [57], [58]), significant levels of behavioural phenotypic plasticity (within-individual variation) allow Adélie penguins to forage efficiently in the highly variable sea ice zone. Although some individuals forage more efficiently than others on average (between-individual variation in intercept or “personality” in the behavioural ecology literature), individual Adélie penguins respond in a similar fashion to variation of SIC in their summer foraging habitat, i.e. there is no between-individual variation in plasticity (also called individual by environment interaction “I×E” [79], [62]). As in previous studies (e.g. [44], [21]), we highlighted sex differences in foraging efficiency. Because Adélie penguins exhibit little size dimorphism, these sexual differences in foraging behaviour may rather arise from a stronger territorial behaviour in males (see [80] for a discussion) than from body size differences.

The level of between-individual variance in average efficiency estimated in our study (11%) is consistent with a recent comprehensive study of provisioning behaviour in house sparrows (*Passer domesticus*) [78]. However, contrary to that study, our analyses did not support significant between-individual variation in how individuals responded to environmental changes. In the context of extremely marked seasonality typical of the Antarctic environment, strong selection forces, coupled with high levels of genetic homogeneity in our study species (perhaps iceberg driven [51]), may prevent individuals from significantly deviating from the population mean response. Alternatively, lack of statistical power could have hampered our ability to detect low or even moderate levels of variance in the response slope. A power analysis [81] allowed us to verify that we were able (power≥0.9) to detect I×E for most values of slope variance (≥0.05) but we cannot rule out the existence of low levels of between-individual variation in the response of Adélie penguins to SIC changes.

### Habitat optimum of Adélie penguins

The conceptual model of the quadratic relationship between Adélie penguin population growth and SIC [82] predict that population growth should increase in polynya-rich, locally lowered SIC (e.g. the Ross Sea; [83]) and decrease in peripheral ice regions (e.g. the north-western Antarctic Peninsula) in accord with real and forecasted total sea ice disappearance [45]. Our current results validate this conceptual model at the level of individual processes in the Ross Sea and provide quantitative estimates for optimum SIC at the mesoscale (tens of kilometers). Indeed, in this species' southernmost breeding habitat, lower SIC in summer will still benefit chick-rearing parents and stimulate population growth as long as overall ice coverage can sustain its ice-dependent Ross Sea prey [84]. According to our results, in the absence of giant icebergs, SIC of about 6% in their foraging area provides optimal foraging conditions during chick-rearing, which coincides with the average SIC experienced during the no-iceberg years (6.6±7.9%). The relatively low preferred SIC (0–12%) highlights the importance of the marginal ice zone (MIZ) for chick-provisioning Adélie penguins [34], [85], [86], [87], [34]. Indeed, local areas of diminished SIC, i.e. polynyas, are responsible for where Adélie penguin colonies are located in most of the high latitude Southern Ocean [34], [85]. In the case of the Cape Crozier colony, penguins forage in the MIZ of the Ross Sea Polynya where they encounter the 6% ice cover. Otherwise, high summer SIC (13–38% or higher) increases foraging trip duration and reduces foraging success as penguins have to walk over extended, continuous stretches of ice, or hop from floe to floe to avoid being crushed or attacked by predators hunting in the leads.

### Effect of extreme climatic events on plastic responses

In the past 30 years, not only have “the climate dice” [4] become more and more loaded (i.e. warmer than usual temperatures, stronger winds and increased precipitation have occurred more frequently, changing the average), but they have also started to roll new numbers (i.e. the frequency of extreme anomalies has increased [4]). In parallel, while the plastic responses of organisms to a gradually changing environment are being increasingly studied, little is known about the effect of extreme events on these responses [31]. A growing body of theoretical work suggests that extreme events could disrupt the plastic response, such that the reaction norm may take any shape in environments that were rarely encountered in Holocene times, therefore inducing non-adaptive plasticity [54], [55], [56]. However, there is still very little empirical evidence to support these findings (but see [88]). Here, our results show that extreme events can provide a natural experiment that reduces the time scale of environmental change [43] and which can show how the shape of the relationship between an environmental variable and a phenotypic trait is modified. On the other hand, such events could become more frequent with the accelerating outflow or in some cases retreat of Antarctic ice shelves [19], thus duplicating the process of rapid ice sheet retreat in the Ross Sea that began 12,000 years ago and stabilized in only the last few thousand years [89]. Such a reversion to frequent ice shelf collapse could significantly complicate our efforts to predict the future effect of environmental changes forecasted over the long term.

By reducing access to prime foraging area, restricting the normal drift of pack ice and decreasing primary productivity [22], giant icebergs constrained the range of variation in Adélie penguin foraging efficiency, essentially uncoupling it from SIC variations and suppressing phenotypic plasticity. The lower value of mean foraging efficiency in years of extreme events also supports theoretical findings that environmentally induced phenotypes have reduced average fitness in extreme environments [54]. Paradoxically, as it remained more or less constant over the range of experienced SIC, the foraging efficiency of Adélie penguins breeding in the presence of giant icebergs became higher on average than for birds breeding in “normal” conditions when SIC was high (>20.6%). These environmental conditions (presence of giant icebergs and SIC>20.6%) were only found in 2002, early in the season, and led to early breeding failures (G. Ballard, unpublished data). Additionally, Lescroël et al. [21] showed that the presence of giant icebergs had the role of revealing individual heterogeneity in foraging ability, i.e. better breeders were more efficient foragers when environmental conditions were harsh. Therefore, it might be possible that relatively high foraging efficiency levels (although lower than under “normal” conditions for most of the experienced SIC range) could be maintained in presence of giant icebergs because of a higher proportion of “high quality” individuals in the breeding population in iceberg years, i.e. “lower quality” individuals skipped breeding or failed earlier in harsh environmental conditions.

The only observed mega-iceberg calving event in the Ross Sea previous to 2001occurred in 1983 [90] but this iceberg did not become grounded in the southern Ross Sea. Whether or not the 2001 calving of icebergs C16 and B15A represent an increasing trend remains to be seen; the outflow of the Ross Ice Shelf has been accelerating owing to a number of climate-related factors [57] and another mega-iceberg, C-19, calved off the Ross Ice Shelf in 2002, but quickly exited the Ross Sea [91]. It appears highly likely that these icebergs could have had similar effects on Adélie penguins of the northern Ross Sea colonies, offshore of which they temporarily grounded and trapped sea ice as they worked their way out of the Ross Sea [91].

While the maximum foraging efficiency of Adélie penguins was significantly lower during years of extreme events, it was still sufficient to raise a chick according to chick growth modelling studies [92]. In that sense, and although giant icebergs also induced increased dispersal [11] and decreased chick production at the population level, this extreme environmental event did not have extreme, long-lasting, ecological consequences for Adélie penguins (see [14] for a discussion of environmental extremes versus ecological extremes). Hence, together with recent evidence of continuing growth of the Ross Sea metapopulation [93], we suggest that, in the absence of any additional anthropogenic disturbance (e.g. industrial fishing, ocean acidification), the Ross Sea may remain a suitable habitat for a large proportion of the Adélie penguin world population across the coming 2–4 decades (after which sea ice cover is predicted to decrease by 5–15% [26]).
